# *Rhodiola crenulata* extract for prevention of acute mountain sickness: a randomized, double-blind, placebo-controlled, crossover trial

**DOI:** 10.1186/1472-6882-13-298

**Published:** 2013-10-31

**Authors:** Te-Fa Chiu, Lisa Li-Chuan Chen, Deng-Huang Su, Hsiang-Yun Lo, Chung-Hsien Chen, Shih-Hao Wang, Wei-Lung Chen

**Affiliations:** 1 Department of Emergency Medicine, Chang Gung Memorial Hospital, Fu-Hsing Street, Kweishan, Taoyuan, Taiwan; 2 Chang Gung University College of Medicine, Wen-Hua 1st Road, Kweishan, Taoyuan, Taiwan; 3 Department of Internal Medicine, Far-east Polyclinics, Yong-Sui Street, Taipei, Taiwan; 4 Division of Biostatistics, Graduate Institute of Epidemiology and Preventive Medicine, College of Public Health, National Taiwan University, Syu-Jhou Road, Taipei, Taiwan; 5 Department of Family Medicin, Chang Gung Memorial Hospital, Fu-Hsing Street, Kweishan, Taoyuan, Taiwan; 6 Department of Emergency Medicine, Taipei Medical University Hospital, Wu-Hsing Street, Taipei, Taiwan; 7 Department of Physical Education, National Taitung University, Chunghua Road, Taitung, Taiwan; 8 Department of Emergency Medicine, Cathay General Hospital, Sec. 4, Jen-Ai Road, Taipei, Taiwan; 9 School of Medicine, Fu-Jen Catholic University, Zhongzheng Road, Xinzhuang, Taipei, Taiwan

**Keywords:** Acute mountain sickness, High altitude illness, *Rhodiola*

## Abstract

**Background:**

*Rhodiola crenulata* (*R. crenulata*) is widely used to prevent acute mountain sickness in the Himalayan areas and in Tibet, but no scientific studies have previously examined its effectiveness. We conducted a randomized, double-blind, placebo-controlled crossover study to investigate its efficacy in acute mountain sickness prevention.

**Methods:**

Healthy adult volunteers were randomized to 2 treatment sequences, receiving either 800 mg *R. crenulata* extract or placebo daily for 7 days before ascent and 2 days during mountaineering, before crossing over to the alternate treatment after a 3-month wash-out period. Participants ascended rapidly from 250 m to 3421 m on two separate occasions: December 2010 and April 2011. The primary outcome measure was the incidence of acute mountain sickness, as defined by a Lake Louise score ≥ 3, with headache and at least one of the symptoms of nausea or vomiting, fatigue, dizziness, or difficulty sleeping.

**Results:**

One hundred and two participants completed the trial. There were no demographic differences between individuals taking *Rhodiola*-placebo and those taking placebo-*Rhodiola*. No significant differences in the incidence of acute mountain sickness were found between *R. crenulata* extract and placebo groups (all 60.8%; adjusted odds ratio (AOR) = 1.02, 95% confidence interval (CI) = 0.69–1.52). The incidence of severe acute mountain sickness in *Rhodiola* extract vs. placebo groups was 35.3% vs. 29.4% (AOR = 1.42, 95% CI = 0.90–2.25).

**Conclusions:**

*R. crenulata* extract was not effective in reducing the incidence or severity of acute mountain sickness as compared to placebo.

**Trial registration:**

ClinicalTrials.gov NCT01536288.

## Background

Acute mountain sickness (AMS) occurs in individuals rapidly ascending to high altitude and it usually results in headache, along with anorexia or nausea, fatigue, dizziness, and insomnia [1]. The number of people travelling rapidly to higher altitudes for work or recreation is rising. As a result of improvements in transportation to these high-altitude regions, AMS has become a significant environmental health issue [2]. The most effective preventive method for AMS—gradual ascent—is usually difficult or impractical for modern international travelers to locations such as Lhasa in Tibet (3650 m) and La Paz in Bolivia (3740 m) [3]. The pathophysiology of AMS is not totally understood but it is apparent that administration of prophylactic treatments is helpful. Historically acetazolamide has been used as a gold standard in treatments to prevent malaise symptoms at high altitudes [1,2,4-7]. However, acetazolamide requires a prescription and has side effects such as paresthesia, dysgeusia, and diuresis [2,8,9]. Some over-the-counter herbal supplements such as *Rhodiola* species, *Ginkgo biloba* and Coca leaf products are widely used [3,5,7,10-17].

Genus *Rhodiola* (family Crassulaceae) has been a valuable medical plant in European countries for thousands of years [10,18,19]. There are more than ninety species worldwide and they are well known for their high antioxidant activities. Different species of *Rhodiola* differ in their content of bioactive components and medicinal use in indigenous regions. To date, most studies focus on the efficacy of *Rhodiola rosea* in treating physical and mental fatigue. Of all the known *Rhodiola* species, *Rhodiola crenulata* (*R. crenulata*) is particularly described in the Pharmacopoeia of China [16] and is considered as the highest in quality. Also, it has been used for treating acute mountain sickness in Tibet since ancient times [20].

Pulmonary alveolar hypoxia, a common phenomenon for non-acclimatized individuals who abruptly relocate to a high altitude, is thought to contribute to the impaired trans-alveolar fluid transport. The excessive fluid that subsequently accumulates in alveoli exaggerates alveolar hypoxia and obstructs the gas exchange process, leading to the pathological progression of high altitude pulmonary edema, the most lethal form of high-altitude illnesses [21,22]. In a rodent model, *R. crenulata* extract exhibited a high antioxidant activity and attenuated pulmonary edema induced by hypobaric hypoxia [20]. Recent studies have shown that Na/K-ATPase plays a key role in alveolar fluid clearance. Both inhibition and knockdown of Na/K-ATPase expression significantly reduced the alveolar fluid clearance in rodent models [23,24]. Two bioactive components of *R. crenulata*, salidroside and tyrosol, were shown to hold antioxidant, anti-depression, anti-fatigue, and anti-inflammatory activities [25-30]. Therefore, the capabilities of *R. crenulata* in preventing the hypoxia-mediated Na/K-ATPase endocytosis and maintaining the integrity of alveolar-capillary barrier and pulmonary sodium transport might be the underlying mechanisms of its effect against pulmonary edema in rodent models [31].

Although *R. crenulata* is widely used to prevent AMS in the Himalayan areas and in Tibet [20,32], but no scientific studies have examined its effectiveness in humans. We conducted a controlled study to investigate the efficacy of *R. crenulata* in AMS prevention. In the clinical trials for *Rhodiola*, most studies were conducted to investigate whether *Rhodiola* could improve the endurance exercise performance or protect against fatigue. The daily dosage of *Rhodiola* extract tested was mostly under 500 mg (ranged between 100–972 mg) [15,18,19]. Regarding the duration of prophylactic medication for AMS, chemical drugs such as acetazolamide could elicit a quick response and were usually administered on the day before or prior to the ascent. In the trials on AMS prevention using prophylactic herbal medication, *Ginkgo biloba* was the one mostly studied. It was often administered for one to five days prior to the ascent [3,11-13]. Considering the onset of AMS after ascent and the compliance of prophylactic medication, a high dose of *R. crenulata* (800 mg daily) was chosen in this study and the subjects were administered with *R. crenulata* for seven days prior to the ascent.

## Methods

### Ethics statement

This study has been approved by the Institutional Review Board (IRB) of Chang Gung Medical Foundation, Linkou Medical Center, Taoyuan, Taiwan. The IRB approved number is "99-3057C" and the topic is titled "Can *Rhodiola Crenulata* Intake Improve Oxygen Saturation and Decrease the Incidence of Acute Mountain Sickness". Subjects were given written information and a verbal explanation concerning the study prior to obtaining the written informed consent for their participation.

### Subjects

We included local Chinese adults aged 23 to 55 years who resided principally at an elevation of 250 m or lower. We excluded those who (1) would not complete the study protocol of two 9-day treatment courses; (2) had prophylactic medication or herbs one month before each ascent (including acetazolamide, sildenafil, tadalafil, dexamethasone, nifedipine, *Rhodiola* species, *Ginkgo biloba*, Eleuthero Root, *Salvia miltiorrhiza,* and sea buckthorn); (3) change in altitude of residence for more than 200 m between ascents; (4) had additional physical training or were scheduled to gain or lose weight; (5) had altitude exposure above 2500 m within three months prior to each ascent; (6) had any history of chronic obstructive pulmonary disease, heart failure, cerebral neoplasm, mania, renal or hepatic insufficiency; or (7) were pregnant or intended to become pregnant during the 3-month study period. After baseline assessment, the subjects were randomly assigned into either sequence.

### Study design, randomization and blinding

This study was a randomized, double-blind, placebo-controlled and crossover trial. Subjects were randomized to 2 treatment sequences, receiving either the *R. crenulata* or the placebo, and then crossed over to the alternate treatment after a 3 month wash-out period. Random numbers were generated by using the computer, using block randomization with a block size of 2 or 4. The random numbers were placed in sealed envelopes, and a serial number was assigned to each envelope according to the sequence of allocation of the randomized number. Each envelope was then opened sequentially, according to the admission sequence of the participants at the study center. The number inside the envelope determined the treatment sequence that each participant was allocated to (*Rhodiola*-placebo or placebo-*Rhodiola*). Both investigators and participants were blinded. One investigator, who was not responsible for any assessment, enrolled all participants and allocated them to treatment. Blinding was maintained until the data analysis was complete.

### Medications

Commercial pharmaceutical grade *R. crenulata* and placebo were packed by Kaiser Pharmaceutical & Biotanico (Tainan, Taiwan). Both were pink soft gelatin capsules (400 mg/capsule) in identical containers and there were no differences in the taste or smell of the capsules. The standardized *R. crenulata* extract (with 2.38% salidroside and 0.44% *p*-tyrosol) was manufactured from the same batch of raw materials [33,34]. The origin of the herb was authenticated by microscopic identification and sequence analysis of the internal transcribed spacer regions. The material was examined for microorganisms, heavy metals, and pesticide according to the accepted standards (good manufacturing practice, GMP) of Taiwan. Each participant received a high dose (800 mg) of *R. crenulata* or placebo (800 mg) daily for 9 days. Beginning 7 days before an ascent, participants were required to inform the investigators by email or telephone every day about the study capsule supply or any adverse effects. If the investigators were not contacted before noon, they would follow up by phone to obtain this information. Moreover, the participants were requested to take capsules every morning of their 2-day mountaineering trip.

### Ascent

**Figure 1:**
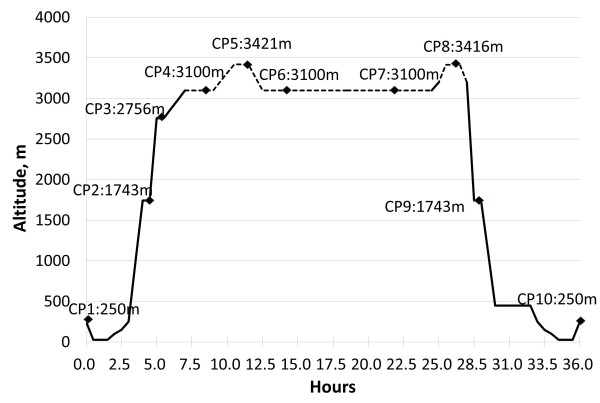
**Mountaineering schedule.** Total of 10 checkpoints (CPs): CP 1, Linkou (250 m); 2, Cingjing Farm (1743 m); 3, Yuanfeng parking lot (2756 m); 4, training camp (3100 m) at noon; 5, East Peak (3421 m) of Hehuan Mountain; 6, training camp (evening); 7, training camp (next morning); 8, Main Peak (3416 m) of Hehuan Mountain; 9, Cingjing Farm and 10, Linkou. Solid line indicates that participants were transported by bus.

To control the ascent rate, participants were transported by bus from an altitude of 250 m to 3100 m (the training camp) within 4 h (Figure 1); they rested for 2 h, hiked 1 km to the East Peak (3421 m, 24°N 121°E) of Hehuan Mountain, Taiwan within 1.5 h, rested 30 min, and descended back to the training camp (3100 m), where they remained overnight. On the second day, all participants were transported by bus to the entrance (3200 m) of the main peak of Hehuan Mountain, and they went hiking along the easy trekking path to the peak (3416 m). After that, they returned to the entrance of the main peak and traveled back to Linkou by bus. There were 10 checkpoints for assessment, namely, Linkou (250 m), Cingjing Farm (1743 m), Yuanfeng parking lot (2756 m), training camp (3100 m), the East Peak (3421 m) of Hehuan Mountain, training camp in the evening and morning, the Main Peak (3416 m) of Hehuan Mountain, Cingjing Farm, and Linkou. During the mountaineering trips, the following variables were controlled: rate of ascent, path, food type, and sleep altitude/environment. The training camp could not accommodate all subjects at once. Thus, the participants were divided into 2 groups in each period, and they ascended separately in less than 10 days apart. The usual snow season of the Hehuan Mountain is between early January and early March. We chose early and mid-December 2010, late March, and early April 2011 as our 4 mountaineering dates. The atmospheric pressure and temperature were recorded at each checkpoint during every trip.

### Measurements

The Lake Louise scoring system (LLS)—a simple and widely accepted tool developed by the Lake Louise Consensus Group [1,35]—was used to assess the AMS grade. The LLS rates 5 symptoms (headache, gastrointestinal symptoms such as nausea and vomiting, fatigue and/or weakness, dizziness and/or light-headedness, and difficulty sleeping) on a self-report questionnaire, with each item graded on a scale from 0 to 3. Thus, the LLS scores can range from 0 (no symptoms or signs) to 15 (the worst rating on each symptom) [12,13,35].

The AMS grade, Lake Louise self-report questionnaire, and pulse oximetry (SpO_2,_ NPB 40, Nellcor, Pleasanton, CA, USA) were completed at 10 checkpoints along the route. Four emergency physicians, familiar with the management of AMS, took care of any discomfort of the participants during the whole course of mountaineering. Participants were not permitted to self-treat their AMS symptoms. Instead, they were told in advance that investigators would provide oral acetaminophen (500 mg) or ibuprofen (400 mg) for headache, meclizine (25 mg) for dizziness or intramuscular prochlorperazine (5 mg) for nausea and/or vomiting.

All subjects had their own mountaineering health passport. It had a list of symptoms related to AMS, advice and precautions to take during mountaineering, a place to record pulse oximetry values, and the Lake Louise questionnaire (to be filled out at each checkpoint). It was also used to record clinical problems and treatment.

### Outcomes

The predetermined primary outcome was the incidence of AMS. It was evaluated at checkpoints 3 to 8 (altitude above 2500 m) or at any time of discomfort after ascent. AMS was defined as LLS score ≥ 3 with headache and at least one of the symptoms of nausea or vomiting, fatigue, dizziness, or difficulty sleeping. Predetermined secondary outcomes included incidence of severe acute mountain sickness (LLS score ≥ 5), incidence of headache and severe headache (defined as a headache score of 2 or 3 on the headache item of LLS), oxygen saturation by pulse oximetry (SpO_2_) at checkpoint 4 (3100 m) and its difference between altitudes 250 m and 3100 m (∆SpO_2_: SpO_2_ measured at checkpoint 1 minus SpO_2_ at checkpoint 4).

### Sample size

The incidence of AMS in placebo group was hypothesized as 36% according to our previous survey [36]. A relative risk reduction of 30% was considered the clinically significant effect in this type of treatment. According to the study design, assuming the absence of carry-over or period effects, a sample size of 133 participants was deemed sufficient to provide a power of 80% with a two-sided alpha level of 0.1 and a probability of discordant pairs of 0.7 to detect an odds ratio for the incidence of AMS of 0.59 between the *Rhodiola* group and the placebo group.

### Statistical methods

Baseline characteristics are presented as mean ± SD (standard deviation) or counts (percentages), as appropriate. For each variable, participants were grouped according to sequence (*Rhodiola*-placebo or placebo-*Rhodiola*) to make the baseline comparison. Comparisons between the two sequences were analyzed by the two-sample independent *t* test for continuous variables and Chi-square test/ Fisher exact test for categorical variables. Carry-over effects and period effects were assessed using the generalized linear models with generalized estimating equations, assuming a logit link function and an unstructured correlation.

If neither a period nor a carry-over effect occurred, paired *t* test or McNemar test was used to test the treatment effect. Otherwise, the generalized linear models with generalized estimating equations were used to analyze the treatment effect, assuming a logit link function and an unstructured correlation for binary outcomes or an identity link function and an unstructured correlation for continuous outcomes, and assuming adjustment for period and carry-over effects in the model. All statistical assessments were evaluated at a two-sided significance level of 0.05. Analyses were performed with SAS software package, version 9.2 (SAS Institute Inc., Cary, NC, USA).

## Results

### Participants

**Figure 2:**
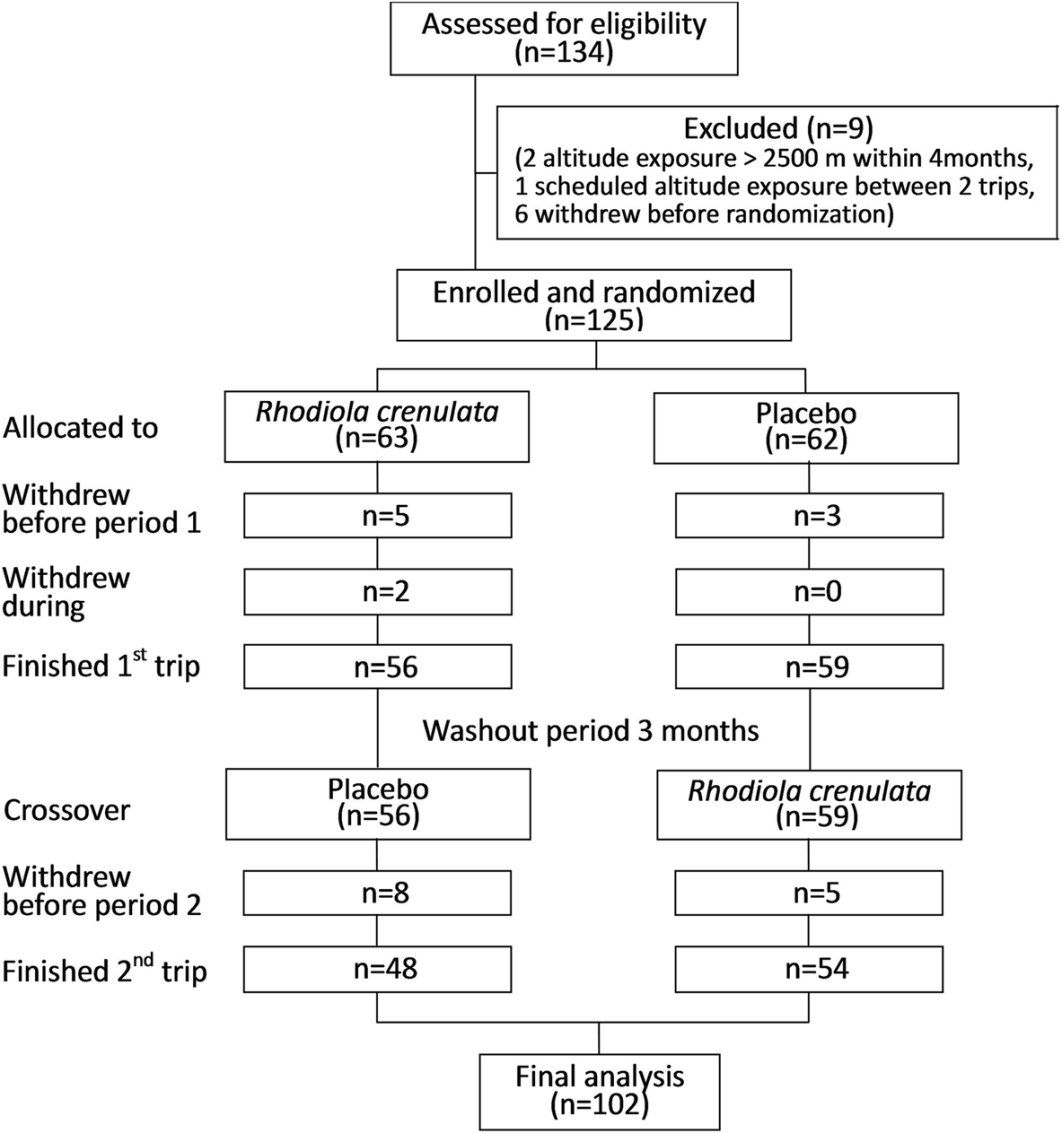
Participant flow diagram.

All 125 participants were recruited to the study in November 2010, and they made two separate trips to Hehuan Mountain: one in early or mid-December 2010 and one in April 2011 or early May. On the second trip, the ascent of one half of the participants was terminated midway (1740 m) because of an unexpected snow storm in late March 2011. Another trip was rescheduled to early May 2011. The trial flow diagram is shown in Figure 2. In all, 23 participants withdrew: 5 from the *Rhodiola*–placebo sequence before the first ascent, 3 from the placebo–*Rhodiola* sequence before the first ascent, 2 from the *Rhodiola*–placebo sequence during the first ascent, 8 from the *Rhodiola*–placebo sequence before the second ascent, and 5 from the placebo–*Rhodiola* sequence before the second ascent. The reasons for withdrawal included interim business trips (n = 4); infections before ascent (n = 4, 3 with upper respiratory tract infection, 1 with leg cellulitis); illness of a girlfriend (n = 1); missed departure due to oversleeping (n = 1); and postponement of the second trip (n = 6 because of work shift problems and 5 due to scheduled travel abroad). One subject in the *Rhodiola*–placebo sequence developed severe vertigo and vomiting at 7 h after ascent during the first trip and was urgently evacuated. Her spouse who also was in the *Rhodiola*–placebo sequence descended with her. The full study protocol was completed by 102 participants. All participants were exposed to the same temperatures, as no statistically significant differences were found between the four ascents (Table 1).

**Table 1:** Ambient temperatures during the four ascents

**Ascents**	**Temperature (°C)**			***P *** **value**
	**Checkpoint 5**	**Checkpoint 6**	**Checkpoint 7**	
Dec. 03, 2010	8	10	9	0.060
Dec. 12, 2010	9	7	7	
Apr. 08, 2011	9	12	12	
May 03, 2011	10	14	12	

Comparison of temperature among the four ascents was assessed by Kruskal-Wallis Test.

### Baseline characteristics

None of the participants (54 women, 48 men; average age, 36.1 ± 10.2 years) went mountaineering regularly, but none avoided outdoor sports. Participants in both sequences (Table 2), *Rhodiola*–placebo and placebo–*Rhodiola*, had similar baseline characteristics (gender, age, body mass index, blood pressure, blood oxygen content, heart rate, mountaineering experience, and AMS history before first period; all *P* > 0.05).

**Table 2:** Baseline characteristics

**Treatment sequences**			
**Characteristic**	***Rhodiola *– placebo**	**Placebo – *Rhodiola***	***P *** **value**
	**(N = 48)**	**(N = 54)**	
Male	23 (47.9)	27 (50.0)	0.83
Age, y	35.8 ± 10.0	36.3 ± 10.4	0.84
BMI, kg/m^2^	22.6 ± 2.9	23.4 ± 3.0	0.17
SpO_2_*, %	98.8 ± 0.9	98.9 ± 1.0	0.60
Heart rate, beats/min	72.5 ± 8.9	72.2 ± 9.6	0.89
Altitude of residence, m	151.7 ± 105.9	152.7 ± 105.9	0.94
History of mountaineering above 3000 m			0.18
Never	23 (47.9)	36 (66.7)	
<10 mountains	20 (41.7)	16 (29.6)	
>10 mountains	5 (10.4)	2 (3.7)	
History of AMS	7 (14.6)	5 (9.3)	0.54

Continuous variables are presented as mean ± standard deviation, and categorical variables are presented as count (percentage).

BMI, Body mass index.

^*^SpO_2_, Oxygen saturation by pulse oximetry at an altitude of 250 m. Heart rate and other demographics were measured during the participant conference before randomization.

AMS, Acute mountain sickness.

### Period effect and carry-over effect

There was a significant difference in the incidence of AMS between period 2 (55.9%; 57/102) and period 1 (65.7%; 67/102) (odds ratio: 0.66, 95% confidence interval: 0.45 ~ 0.98). A period effect (*P* = 0.040) was observed for the primary outcome, but no carry-over effect was found (*P* = 0.877).

### Major outcome measures

**Figure 3:**
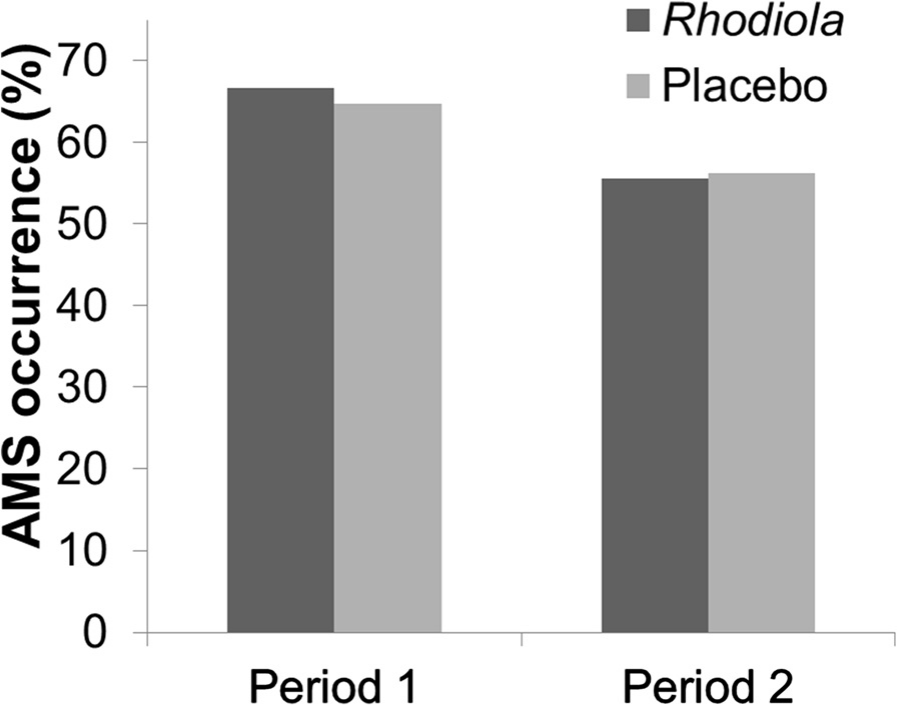
**AMS occurrence in 2 periods.** The incidence of AMS was 65.7% (67/102) in period 1 and 55.9% (57/102) in period 2 (OR: 0.66, 95% CI: 0.45 - 0.98). There were no between-group differences in either period.

The incidence of AMS was the same among participants who received *Rhodiola* extract or placebo (all 62/102, 60.8%). Furthermore, the AMS incidences in the *Rhodiola* and placebo groups were 66.7% and 64.8% in period 1, compared with 55.6% and 56.3% in period 2, respectively (Figure 3). After adjusting for the period effect, the risk of AMS in participants taking *Rhodiola* was 1.03 times that of participants taking placebo, and no significant difference was found between the *Rhodiola* and placebo groups (95% confidence interval: 0.69–1.52; Table 3). About half of the participants (50/102) had trip 2 rescheduled because of a snowstorm, meaning that those taking *Rhodiola* had double the planned dosage*.* Even for these participants, the AMS incidence in the *Rhodiola* group (56.0%, 14/25) was not reduced compared to the placebo group (48.0%, 12/25).

**Table 3:** Outcomes

**Outcome**	***Rhodiola***	**Placebo**	**Odds ratio/difference**	**Adjusted**
	**(N=102)**	**(N=102)**	**(95% CI)**	***P *** **value**
**Primary endpoint**				
AMS^a^	62 (60.8)	62 (60.8)	1.02 (0.69, 1.52) ^††^	0.90
**Secondary endpoint**				
Severe AMS^b^	36 (35.3)	30 (29.4)	1.42 (0.90, 2.25) ^††^	0.13
Headache	81 (79.4)	78 (77.5)	1.17 (0.75, 1.83) ^††^	0.48
Severe headache^c^	33 (32.4)	30 (29.4)	1.16 (0.71, 1.89) ^††^	0.55
SpO_2_, %^d^	88.6 ± 3.9	88.6 ± 4.3	–0.13 (–0.93, 0.66) ^‡‡^	0.74
∆SpO_2_, %	9.6 ± 3.8	9.5 ± 4.2	0.16 (–0.65, 0.97) ^‡‡^	0.70
Pulse rate, /min^e^	99.2 ± 14.6	99.8 ± 14.2	–0.30 (–2.61, 2.02) ^‡‡^	0.80
**Other clinical measures**				
AMS symptoms score^f^				
Headache	1.17 ± 0.81	1.13 ± 0.83	0.05 (-0.10, 0.20) ^‡‡^	0.50
Dizziness	0.87 ± 0.82	0.79 ± 0.79	0.09 (-0.06, 0.25) ^‡‡^	0.29
Weakness	0.94 ± 0.81	0.99 ± 0.84	–0.02 (–0.17, 0.13) ^‡‡^	0.81
Vomiting	0.52 ± 0.70	0.41 ± 0.62	0.12 (–0.01, 0.25) ^‡‡^	0.08
Sleep	1.45 ± 0.99	1.55 ± 0.96	–0.07 (–0.29, 0.14) ^‡‡^	0.49
LLS score	3.84 ± 2.49	3.77 ± 2.54	0.19 (-0.27, 0.55) ^‡‡^	0.51

For each outcome, data were grouped into two datasets (one for *Rhodiola* and one for placebo) to create a data summary. Categorical outcomes are presented as count (percentage), and continuous outcomes as mean ± standard deviation. The treatment effect is presented in the column "Odds ratio/difference (95% CI)". Odds ratio is used for categorical outcomes and defined as *Rhodiola* versus placebo, while difference is used for continuous outcomes and defined as *Rhodiola* minus placebo. The generalized linear models with generalized estimating equations method, assuming logit/ identity link as appropriate and unstructured correlation, was used to obtain the results after adjustment of period effects.

^a^ Lake Louise score (LLS score) ≥3 with headache and at least one other symptom was defined as AMS. At each period, occurrence of AMS during checkpoints 3 to 8 was defined as AMS.

^b^ AMS and LLS score ≥5.

^c^ Defined as headache score of >1 on the headache item of LLS (ascending scale of 0–3 for severity).

^d^ SpO_2_, oxygen saturation by pulse oximetry (SpO_2_) at checkpoint 4 (3100 m).

∆SpO_2_, the difference between checkpoints 1 and 4 (SpO_2 at checkpoint 1_ – SpO_2 at checkpoint 4_).

^e^ Pulse rate was measured at checkpoint 4 (3100 m).

^f^ All symptoms in AMS are defined as the worst score in the interval between checkpoints 3 and 8.

†† odds ratio.

‡‡ difference.

After adjustment for period effect, between-group differences in the incidence of severe AMS, headache, severe headache, SpO_2_ level and pulse rate at checkpoint 4, and ∆SpO_2_ level were not significant (all *P* > 0.05) (Table 3).

### Other outcome measures and adverse events

There were no significant differences between *Rhodiola* groups and placebo groups for the comparison of the worst AMS symptom scores and highest LLS scores in the interval between checkpoints 3 and 8 (all *P* > 0.05) (Table 3). The number of subjects requesting medication for headache/dizziness/nausea (vomiting) was about the same in both groups (36/20/4 vs. 35/20/3 for *Rhodiola* and placebo respectively).

During the 7-day period prior to ascent, adverse events were rare. All were rare, mild and lasted less than two days; therefore, no participant needed to stop taking the study drugs prior to ascent (Table 4).

**Table 4:** Adverse events in groups receiving prophylactic agents for AMS

**Adverse event**	***Rhodiola *** **(n = 102)**	**Placebo (n = 102)**
Difficulty in falling asleep	1 (0.98)	1 (0.98)
Light sleep	3 (2.94)	1 (0.98)
Dizziness	2 (1.96)	0 (0.00)
Drowsiness	2 (1.96)	0 (0.00)
Pruritus	1 (0.98)	1 (0.98)
Dry hand	1 (0.98)	0 (0.00)
Abdominal distension	1 (0.98)	0 (0.00)
General soreness	1 (0.98)	0 (0.00)
Dry mouth	0 (0.00)	3 (2.94)
Headache	0 (0.00)	2 (1.96)
Palpitation	0 (0.00)	2 (1.96)
Flushed face	0 (0.00)	1 (0.98)
Increased urination	0 (0.00)	1 (0.98)

Data are presented as count (%).

### Sensitivity analysis

**Figure 4:**
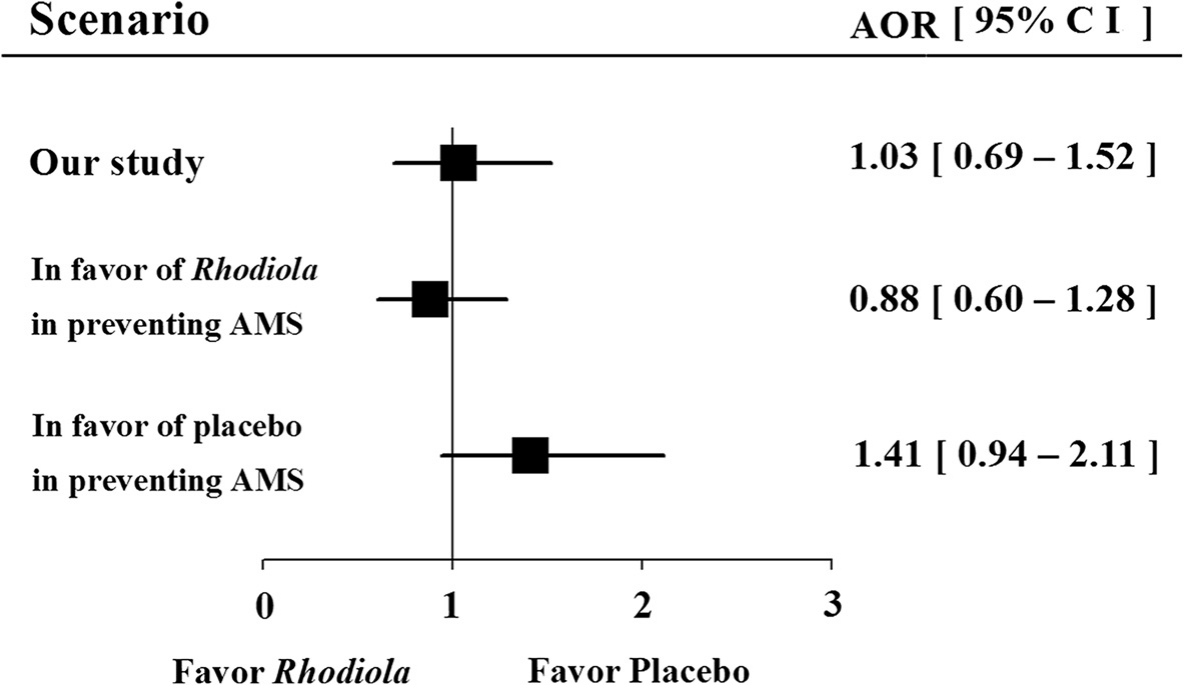
**Sensitivity analysis.** Data was presented as adjusted odds ratio (AOR) and 95% confidence interval (CI). Comparison between Rhodiola arm and placebo arm was analyzed by generalized linear models with generalized estimating equations. Regardless of any extreme scenarios, *Rhodiola* was not effective in preventing AMS. *: significant difference statistically.

In total, there were fifteen subjects who attended mountain climbing during period 1 but were absent during period 2 (Figure 2). To evaluate the impact of dropout on the efficacy for the *Rhodiola* arm compared to the placebo arm, two scenario analyses were carried out in order to fill in the missing values for AMS occurrence. In the first scenario, the missing values were assumed to be in favor of *Rhodiola* in preventing AMS. The odds ratio was 0.88 and 95% confidence interval contains 1 (0.6 - 1.3, *P* = 0.49). In the second scenario which is in favor of the placebo in preventing AMS, and odds ratio was 1.4 and 95% confidence interval contains 1 (0.94 - 2.11, *P* = 0.10). In either scenario, *Rhodiola* was not effective in preventing AMS (Figure 4).

### Assessment of blinding

After the second trip, all participants were asked to guess the trip in which they had taken *Rhodiola*; 49% (50/102) guessed correctly, indicating that the participants identified the treatment period during which they received *Rhodiola* or placebo only by chance.

## Discussion

*R. crenulata* extract was not effective in reducing the incidence or severity of AMS when compared with placebo and failed to show a protective benefit for any outcome measure examined. Statistical analyses showed that there was a period effect but no carry-over effect. AMS occurred significantly less frequently on the second mountaineering trip (65.7% vs. 55.9%; *P* = 0.040), regardless of treatment. Since most participants (58%) had no high-altitude mountaineering experience prior to the first trip, the first trip experience helped them adapt to conditions on the second trip in period 2. Inadequate high-altitude mountaineering experience was proven to be an independent risk factor for AMS [36-39]. In Gaillard’s study, the understanding and awareness of AMS among trekkers reduced the incidence of AMS [40]. Also, Vardy et al. suggested that AMS is less likely to take place with a correct awareness of its symptoms and prevention [41]. In our study, the participants filled the Lake Louise self-report questionnaire at 10 checkpoints during mountaineering, so they had a comprehensive awareness of high altitude experience and AMS symptoms after the first period of mountaineering. They also shared experiences and methods on AMS prevention, such as avoiding high impact activity at high altitude. Therefore, the participants had a significantly superior awareness of symptoms and prevention for AMS in the second period than in the first period of mountaineering. Also, no period effect was observed among the experienced participants in our study. Thus, this phenomenon could be classified as a learning effect. The weather, warmer during period 2, could be the other explanation for the period effect. Our trial was designed conservatively to avoid any possible bias against *R. crenulata*: we used a high dose of this agent, a longer period of drug preloading, and a steep ascent profile from the sea level. During the mountaineering trips, the following variables were controlled: rate of ascent, path, food type, and sleep altitude/environment.

In the present study, the environmental and medical resources available to study AMS were limited. We designed our study as a crossover trial for a key reason. Because each participant serves as his or her own control, it skillfully controls for the inherent individual susceptibility to AMS. Each person has his or her own personal tolerance for altitude change, and consequently, we calculated the percentage change of outcomes from his or her baseline to evaluate the effect of intervention or association with possible factors in parallel design. Using crossover design enabled us to measure the effect of intervention exactly, regardless of individual susceptibility. This design also had the benefits of increasing statistical power, given the limited sample size, and efficient control of the influence of confounding covariates.

Our study had several limitations. First, only 102 of the original 125 participants completed both trips. However, the statistical analyses were still adequately powered for three reasons: (1) the incidence of AMS was higher than what we had hypothesized using data generated in our previous survey [36]. This was because most of our participants were inexperienced mountaineers, and their rate of controlled ascent was significantly higher than that in the previous study (4 h vs. 1–2 days). The participants were requested not to take acetazolamide or other possible prophylactic medications used by trekkers before mountaineering in previous surveys. (2) The sample size was estimated using the assumption that *Rhodiola* would reduce the incidence of AMS by 11% (36% to 25%) compared to placebo, but the results showed no difference between taking *Rhodiola* and placebo. Based on the data obtained in the present study, a sample of infinite size would be required to prove that *Rhodiola* had a significant prophylactic effect. (3) The sensitivity analysis showed that *Rhodiola* was not effective in preventing AMS regardless of the scenarios proposed (Figure 4). The second limitation is that half of the participants had a rescheduled second trip and they therefore took the study medication (or placebo) for 7 days before ascent and then on the first morning of ascent, for 8 days. Although they did not reach an altitude where AMS might be expected, they took study medication for 8 out of 9 days, and then they began medication once again 4 weeks later. Although these participants took double dosage of *Rhodiola*, their AMS incidence was not reduced. The third limitation is that the *Rhodiola* extract purified from the rhizome of *R. crenulata* was used to assure high concentration of active ingredients (2.38% salidroside and 0.44% *p*-tyrosol) in this study. However, the active ingredients for preventing AMS could have been lost during extraction and purification. Many Chinese tourists drink a decoction of *Rhodiola* rhizome to prevent AMS. The results of the present study do not exclude the possibility that this *R. crenulata* decoction may have a preventive effect on AMS. Last, only one dosage and one *Rhodiola* species were tested. Higher dosage or extracts from other species, such as *Rhodiola rosea*, *Rhodiola kirilowii*, *Rhodiola sachalinensis,* and *Rhodiola algida,* might be effective.

Studies have investigated different pharmacologic interventions to prevent AMS. Acetazolamide is effective for the prevention of AMS but it may be associated with paresthesias. Sumatriptan and gabapentin are beneficial but require further study [5]. Antioxidants magnesium, *Ginkgo biloba* and Coca leaf products were not efficacious [5,17]. In the Himalayan region, China, and Andean region of South America, using traditional applications of herbs for anti-high altitude illness is more common than using doctor prescription. For example, Coca leaf products were used more often among 62.8% of the surveyed travelers than acetazolamide prophylaxis (16.6%) for prevention, or other non-pharmacologic measures [17]. Similar in China, 7% of 247 Chinese travelers on Qinghai-Tibet railroad took *Rhodiola* as a prophylaxis for AMS, but none used acetazolamide or dexamethasone, except one who had asthma [32]. It was shown that coca leaf products did not prevent AMS, but increased the chance of AMS occurrence. Therefore, complementary and alternative medicines are omnipresent and popular, and should not be dismissed as "non-medications".

Our study is the first randomized, double-blind and controlled study of *R. crenulata* extract for AMS prophylaxis in humans though the current data does not demonstrate any prophylactic effects. Further studies will be needed to demonstrate the efficacy of *R. crenulata* decoction or other *Rhodiola* species in AMS prevention.

## Conclusions

This randomized double-blind placebo control crossover trial demonstrated that the *R. crenulata* extract was not effective in reducing the incidence or severity of AMS. *R. crenulata* extract should not be recommended as a prophylactic for AMS.

## Competing interests

All authors have no competing interests.

## Authors’ contributions

TFC is responsible for the study design, performing research, data interpretation and drafting of the article. LLC is responsible for the study design, statistical analysis and drafting of the article. DHS is responsible for revising the article and statistical expertise. HYL and CHC are responsible for collection and assembly of data. SHW and WLC both make great contribution to conception and design, critical revision of the article for important intellectual content, interpreting results and revising manuscript. All authors read and approved the final manuscript.

## Pre-publication history

The pre-publication history for this paper can be accessed here:

http://www.biomedcentral.com/1472-6882/13/298/prepub
